# Typhoid in a Kenyan Village: Its Impact, Its Prevention

**DOI:** 10.4269/ajtmh.18-0537

**Published:** 2018-11

**Authors:** Kenneth Simiyu, Leslie Jamka

**Affiliations:** Center for Vaccine Development and Global Health, University of Maryland School of Medicine, Baltimore, Maryland

It is early morning in the Village of Tongaren, a village where I grew up, 15 km from Kitale town in western Kenya. There, on my annual vacation from the United States, where I live and work as Program Director for the Typhoid Vaccine Acceleration Consortium (TyVAC), I meet 8-year old Nanjala walking with her mum to the local health center. I stop and give them a ride; her mother tells me Nanjala has had fever and abdominal cramps for 3 days. The diagnosis at the local health center showed the likely cause, typhoid fever. Nanjala and her mum, informed of the diagnosis by phone, were on their way back to the health center, a distance of 5 km, to receive medication. After dropping them off, I got to thinking about the tragedy that is typhoid.

Typhoid inflicts a significant public health burden in Kenya. The Global Burden of Disease estimates that in 2016, Kenya had 97,762 typhoid cases, 62% among children aged less than 15 years; and 1,075 typhoid deaths, 66% among children aged less than 15 years.

As a child, typhoid was one of the most common ailments in my village. It was often associated with playing in the rain or water. Like in many rural communities, there were no laboratories to carry out blood tests and diagnosis is based on clinical symptoms. Quite often, a child sick with a fever was first given anti-malaria drugs. If there was no improvement, typhoid was the next suspect and the health-care worker prescribed antibiotics for treatment.

I have been away from my village for more than 30 years—a village I left to pursue further studies first in Nairobi and then in the United States and Canada. Working in the public health sector, specifically with typhoid, I am immersed with the disease, its impact, and most importantly, its prevention. Typhoid disproportionately impacts children and low-income populations in Asia and sub-Saharan Africa. If left untreated, it can cause short- and long-term complications. Improved water quality, sanitation, and hygiene are the major ways to disrupt transmission; However, until these investments are realized, vaccination is an important and effective way to reach children most at risk for this devastating disease.

Typhoid fever is caused by the bacterium *Salmonella enterica* serotype Typhi. Although contaminated food and water are the major causes of typhoid, a range of other factors have been associated with outbreaks in endemic settings such as poor sanitation, close contact with typhoid cases or carriers, proximity to contaminated water, flooding, personal hygiene, and densely populated areas. Unfortunately, these were inevitable conditions surrounding my life in Kenya. In addition, climate variables such as rainfall, vapor pressure, and temperature intensify the risks of typhoid transmission and distribution of typhoid infection in human populations.

According to the latest Kenya Demographic and Health Survey, 66.9% of Kenyans have an improved water source, but less than half the rural population in Kenya has access to safe drinking water. The situation is no different in my village where most people still rely on water from unsafe and contaminated sources including the river and nearby streams. In Tongaren, most women have to trek more than a kilometer to fetch drinking water. The same water is used by livestock. This water is often contaminated by human sewage because of the lack of proper sanitation. Less than 25% of the population in Kenya is using an improved sanitation facility. The situation in rural areas like Tongaren is even worse; perhaps less than 15% have improved sanitation.

Poor sewage disposal, often next to waterways, leads to runoff that eventually ends up in rivers and ponds. Water drawn from such rivers and ponds is rarely treated because of a lack of awareness and, to some extent, poverty and scarcity of fuelwood to boil water. Although it is likely that most water is contaminated at the source, as shown in many studies conducted in Kenya, there is often re-contamination during collection, storage, and use at home. In the village, residents typically collect water from rivers in 20-L plastic containers and then store water in the house in large pots for several days. A cup or tin is frequently dipped into the pot to draw drinking water. Multiple people often use the cup, which is rarely cleaned, thus, introducing contaminants, chief among them bacteria such as *Escherichia coli* and typhoid-causing *Salmonella*, into the water.

It is likely that Nanjala contracted typhoid from contaminated water, but this is not the only possible source. Contaminated food can also be a source of typhoid. Nanjala and her friends, like many other school children her age, often buy their lunch from food vendors. This food is often contaminated during preparation, handling, and storage where fruits and vegetables are not properly washed. Street food has been identified as a major cause of typhoid outbreaks in countries including Uganda, Zimbabwe, and India.

Nanjala is among the nearly 12 million cases and more than 128,000 deaths due to typhoid worldwide. Unfortunately, it is young children like Nanjala, and adolescents, who are most affected by typhoid. Typhoid cannot be wished away and there are no home remedies, which is why prevention and proper treatment are so important. Typhoid can affect vital body organs and can be fatal if left untreated.

The effects of typhoid in children like Nanjala extend beyond ill health. It translates into absenteeism from school and lost productivity for caregivers who have to take their children to health facilities, and lost wages for casual workers, the most affected segment of adults, who stay at home to take care of the sick. There is also further economic hardship as precious resources are used to purchase medicine, something that poor families, who are the most vulnerable, can ill afford.

Prevention of typhoid can occur at both the community and individual level. At the community level, prevention includes health awareness programs, treatment of water, and provision of adequate sanitation. At the individual level, personal hygiene is key; one needs to always wash their hands before eating. It is also important to ensure raw food is washed with clean water.

There is great news for children like Nanjala—a new typhoid conjugate vaccine (TCV) has recently been approved by the World Health Organization (WHO). In April 2018, the WHO officially recommended that typhoid-endemic countries introduce TCV in a single dose for infants and children older than 6 months, accompanied by catch-up vaccination campaigns for children aged up to 15 years, where feasible. The WHO recommendation followed a review of evidence on TCV by the Strategic Advisory Group of Experts (SAGE) on immunization in October 2017. The SAGE considered data on vaccine safety, efficacy, feasibility, and affordability, as well as growing rates of drug-resistant typhoid. The WHO position paper emphasizes the importance of using TCV to control endemic and epidemic typhoid.

TyVAC is leading global efforts on typhoid and the introduction of TCVs. Expanded use of TCVs has the potential to reduce the need for antibiotics, slow further emergence of drug-resistant typhoid strains, and save lives. Hopefully, countries will take advantage of this opportunity to combat typhoid and improve the lives of children like Nanjala.

Before I left the village and headed back to the United States, I made a point of touching base with Nanjala and her mum. I provided them with information that would help them avoid getting typhoid, including the need to properly boil water before drinking it and also to always wash their hands before eating. In addition to specific advise to Nanjala, I would like to urge the Ministry of Health of Kenya authorities to take advantage of the availability of the TCV and introduce the vaccine in the country and help save lives.

